# Minutes of the Ninth Annual Meeting of the Kentucky State Dental Association

**Published:** 1879-10

**Authors:** 


### Proceedings of Societies.

### MINUTES OF THE NINTH ANNUAL MEETING OF THE KENTUCKY STATE DENTAL ASSOCIATION, HELD IN LOUISVILLE, JUNE 3, 4, AND 5, 1879.

The Association convened on Tuesday, June 3, at half past two o'clock p. m., in the lecture room of the Reformed Episcopal Church. Dr. F. Peabody, the president, in the chair. After prayer by Rev. J. H. Heywood, the minutes of the last meeting as printed were approved.

On motion of Dr. A. O. Rawls, physicians and visiting dentists were invited to attend, and the courtesies of the floor extended to them.

On motion of Dr. W. H. Goddard, Drs. J. Taft, J. S. Cassidy, and C. E. Dunn, were appointed a committee to draft suitable resolutions on the death of Prof. J. H. McQuillan.

The President, in the absence of Dr. J. T. McMillan, of the Board of Censors, and the position of chairman of said committee being rendered vacant by the election to the presidency of Dr. Peabody, appointed Drs. C. E. Dunn and B. Oscar Doyle to fill the vacancy.

The Board of Censors recommending W. N. Alsop, D. D. S., of Louisville, and Phillip T. Dedman, D. D. S., of Lebanon, were unanimously elected active members of the Association.

The President appointed Dr. C. G. Edwards to fill the vacancy in the Executive Committee, caused by the absence of Dr. J. T. McMillan. Adjourned to meet at half past eight o'clock the following morning.

SECOND DAY—MORNING SESSION.

The President, Dr. F. Peabody, called the meeting to order. E. C. Canine, D. D. S., of Hope, Ind, on recommendation of Board of Censors, was unanimously elected an honorary member of the Association.

On motion, the first and second subjects—Anatomy and Physiology—were passed temporarily, and the third subject, Pathology—"Secondary Dentine, Osseous Pulps, Exostosis, Action and Effects of Dead Teeth," was taken up and discussed by Drs. A. O. Rawls, J. B. Kidd, and W. M. Herriott, discussion passed on special order. The President then read his address, which was received with marked attention and applause by the members. The address was referred to a committee, consisting of Drs. Rawls, Hooper and Dedman, to select such portions as might be deemed advisable to submit to the special action of the Association.

Dr. H. A. Smith, moved that the subjects of Pathology, Histology and Physiology, the latter including "The Provisions of Nature for the Protection of Exposed Pulp, be discussed in union. Motion accepted, and discussion participated in by Drs. Peabody, Doyle, H. A. Smith, Taft, Herriott, Rawls and Kidd.

The committee on President's address reported, calling special attention to that portion which refers to dental education, and the want of interest among some of our members, and among the dentists generally throughout the State. Report accepted.

Dr. W. M. Herriott of Indiana, extended a cordial invitation to the Association to attend the next meeting, 24th inst., of the Indiana State Dental Association at Indianapolis. Adjourned till two P. M.

SECOND DAY—AFTERNOON SESSION.

Meeting called to order by the President. A letter from Dr. J. T. McMillan was read, expressing great disappointment at his inability to attend on account of sickness in his family.

Dr. Hooper, read an interesting paper on "The Provisions of Nature for the Protection of Exposed Pulp." Discussed by Drs. Taft, H. A. Smith and Rawls.

The first subject on the programme which was passed temporarily, viz: "Do the Nerves of Extracted Teeth Unite in Replanting?" was then taken up and discussed by Drs. Edwards, Herriott, Rawls and Taft. The second section of the subject, "What are the conditions of the alveolar process, when there is abscess on the tooth?" was discussed by Drs. Taft, H. A. Smith, Herriott, Edwards, Peabody and Rawls.

The following preamble and resolutions were then read, and approved.

*Whereas:* This Association has learned with deep sorrow, of the recent death of Prof. J. H. McQuillan, of Philadelphia, whose reputation as a scientist was world-wide; a gentleman whose genial qualities rendered him personally dear to many of us, and whose successful efforts in the cause of dental education, made him a shining light in our profession, his premature loss is beyond repair. Therefore:

*Resolved*, that the members of the Kentucky State Dental Association, sincerely sympathize with the afflicted family, and his colleagues of the Institution with which he was connected.

*Resolved:* That a copy of the above preamble and resolution be sent to the family of deceased, to the Faculty of the Philadelphia Dental College, and recorded in the Minutes of this Association.

J. TAFT.
J. S. CASSIDY.
C. E. DUNN.

The Subject of Chemistry. "The Different Agents that Produce Disnitegration of Enamel and Dentine, and Their Mode of Action," was discussed by Drs. Cassidy, H. A. Smith and Rawls. Adjourned to eight o'clock, P. M.

SECOND DAY.—EVENING SESSION.

Association called to order promptly by the President.

On motion of Dr. A. W. Smith, a committee, consisting of Drs. A. W. Smith, Peabody and Rawls, was appointed to prepare a proper handbook on Dental Hygiene.

Discussion on Oral Chemistry resumed by Drs. A. W. Smith, Cassidy, Canine, H. A. Smith, Edwards and Taft. Subject passed. "The therapeutic action of remedial agents upon diseased conditions that cause absorption of the alveolar process and gums," discussed by Drs. Peabody, Doyle, Taft and Edwards.

Adjourned till next day.

THIRD DAY.—MORNING SESSION.

Association called to order at nine a. m. by the President, Dr. Peabody, and the discussion on Therapeutics resumed by Drs. Canine, Peabody, Taft, Doyle, Edwards, Rawls, H. A. Smith, Cassidy and A. W. Smith. Subject passed. On motion the officers were authorized to procure twelve blank diplomas for the Association. Report of Executive Committee on the Treasurer's Report received and adopted.

Subjects of Operative Dentistry and Mechanical Dentistry passed, and on motion the election of officers ordered, the following being the almost unanimous result:

President, J. S. Cassidy.

Vice-President, J. Hooper.

Secretary, C. G. Edwards.

Treasurer, J. F. Canine.

A. W. Smith was elected a member of the Executive Committe, J. B. Kidd was elected a member of the Board of Censors, and F. Peabody was elected a member of the Board of Examiners.

On motion the next place of meeting was considered and Richmond, Ky., was unanimously selected.

All bills recommended by the Executive Committee were ordered paid.

Adjourned till two o'clock p. m.

### THIRD DAY—AFTERNOON SESSION.

Association called to order by the President.

The Executive Committee reported committees on subjects for discussion at next meeting as follows:

Anatomy—B. O. Doyle and J. B. Kidd.

Physiology—W. W. Justice and J. Hooper.

Pathology—F. Peabody and C. G. Edwards,

Chemistry—J. S. Cassidy and F. L. Klingman.

Therapeutics—C. E. Dunn and A. O. Rawls.

Histology—J. T. McMillan and W. N. Alsop.

Operative Dentistry—A. W. Smith and J. F. Canine.

Mechanical Dentristry—W. G. Redman and P. T. Dedman.

### MISCELLANEOUS.

Drs. P. T. Dedman, W N. Alsop and J. Hooper, were selected delegates to the American Dental Association.

Dr. C. E. Dunn offered the following amendment to the By-Laws, which are to be acted upon at next meeting: Law IV to read, "All graduates for active membership two dollars" instead of three dollars. "Non-graduates for active memberhsip eight dollars" instead of twenty dollars. Law V, sec. 1st, amend so as to read "two dollars" instead of three dollars.

Dr. Goddard presented the following resolutions, which were adopted:

*Resolved*, That a vote of thanks be, and are hereby, tendered to the Trustees of the Reformed Episcopal Church of this city for the use of their lecture room for our meeting.

*Resolved*, That the thanks of this Association be tendered to the daily press of Louisville for publishing our proceedings.

*Resolved*, That the thanks of this Association be, and are hereby, tendered to our retiring President, for the courtesy, gentlemanly and parliamentary manner in which he has performed his duties; also to the retiring Secretary.

*Resolved*, That the thanks of the Association are heartily extended to our brother Dr. Chas. E. Dunn for so kindly and ably reporting our proceedings.

The officers elect were then installed.

The retiring President thanked the members for their uniform courtesy during the session, and hoped that he was leaving the chair as honorably as he had found it, and that his successor, at the end of his term, could say the same. The President-elect was conducted to the chair by Drs. Goddard and Taft. He made a few remarks appropriate to the occasion, claiming with some feeling, that no class of men were more worthy of the gratitude and respect of intelligent people than those who constituted the various dental associations.

Adjourned to meet at Richmond, Ky., on the first Tuesday in June 1880.

